# Preservation of optic nerve structure by complement inhibition in experimental glaucoma

**DOI:** 10.1007/s00441-020-03240-7

**Published:** 2020-07-17

**Authors:** Caroline J. Gassel, Sabrina Reinehr, Sara C. Gomes, H. Burkhard Dick, Stephanie C. Joachim

**Affiliations:** Experimental Eye Research Institute, University Eye Hospital, Ruhr-University Bochum, In der Schornau 23-25, 44892 Bochum, Germany

**Keywords:** Glaucoma, Optic nerve, Complement system, Microglia, Complement inhibition

## Abstract

Glaucoma is characterized by a progressive damage of the retina and the optic nerve. Despite a huge research interest, the exact pathomechanisms are still unknown. In the experimental autoimmune glaucoma model, rats develop glaucoma-like damage of the retina and the optic nerve after immunization with an optic nerve antigen homogenate (ONA). An early activation of the complement system, even before optic nerve degeneration, was reported in this model. Here, we investigated the effects of a monoclonal antibody against complement factor C5 on optic nerves. Rats were immunized with ONA and compared to controls. In one eye of some ONA animals, the antibody against C5 was intravitreally injected (15 μmol: ONA + C5-I or 25 μmol: ONA + C5-II) before immunization and then every 2 weeks. After 6 weeks, optic nerves were processed for histology (*n* = 6/group). These analyses demonstrated that the intravitreal therapy reduced the depositions of the membrane attack complex compared to ONA animals (ONA + C5-I: *p* = 0.005; ONA + C5-II: *p* = 0.002). Cellular infiltration was significantly reduced in the ONA + C5-I group (*p* = 0.003), but not in ONA + C5-II tissues (*p* = 0.41). Furthermore, SMI-32 staining revealed that neurofilament was preserved in both treatment groups compared to ONA optic nerves (both *p* = 0.002). A decreased amount of microglia was found in treated animals in comparison to the ONA group (ONA + C5-I: *p* = 0.03; ONA + C5-II: *p* = 0.009). We observed, for the first time, that a complement system inhibition could prevent optic nerve damage in an autoimmune glaucoma model. Therefore, complement inhibition could serve as a new therapeutic tool for glaucoma.

## Introduction

Glaucoma is one of the leading causes of blindness worldwide (Flaxman et al. 2017; Tham et al. 2014). Until now, major parts of the pathogenesis of this neurodegenerative disease are still unknown. In particular, normal-tension glaucoma (NTG) puzzles researchers. The currently available therapies, comprising local medication and surgical methods, are often unsatisfactory and have considerable side effects (Gupta and Chen 2016). Therefore, glaucoma, especially NTG, still represents a challenge for the clinician ophthalmologist, making further research in this field necessary. It is a multifactorial disease, where an elevated intraocular pressure (IOP) is the major risk factor. Nevertheless, some patients with primary open-angle glaucoma (POAG) show an IOP within the statistically normal range, known as NTG. The proportion of NTG among all POAG varies depending on the population observed. In a South African study, NTG constituted about 57% of all POAG cases (Rotchford and Johnson 2002), whereas in Caucasians the percentages are lower, with values between 30 and 39% (Bonomi et al. 1998; Dielemans et al. 1994; Klein et al. 1992). In Asian epidemiologic studies, NTG constituted the majority of open-angle glaucoma (Cho and Kee 2014, He et al. 2006, Iwase et al. 2004, Kim et al. 2011).

As most glaucoma medication focuses on IOP reduction, adequate therapeutic options for NTG patients are lacking. Thus, further studies are required to identify pathogenetic hallmarks in NTG and develop new treatment approaches.

Research on the field of glaucoma also points to the immune system as a central pathogenetic playmaker (Grus et al. 2008; Tezel and Wax 2004; Wax 2011). Findings from both clinical and basic-scientific studies reveal the relevance of immune-mediated processes in this disease. For instance, altered antibody patterns were detected in glaucoma patients (Boehm et al. 2012; Joachim et al. 2007). Also, human retinae with glaucomatous damage exhibit IgG antibody depositions in their ganglion cell layer (Gramlich et al. 2013). The complement system, as a part of the innate immune system, could possibly interact with those antibodies. Accordingly, an increased expression of several complement factors in retinal protein samples of glaucoma patients was noted. These include proteins from the lectin and classical pathway of complement activation. Complement factor H, a complement regulatory protein, is downregulated in human glaucoma (Tezel et al. 2010). Furthermore, in the aqueous humor of POAG patients, increased levels of the complement protein C3 were detected (Liu et al. 2020). This knowledge led to the assumption that an excessive complement attack resulting from a dysregulation could cause neuronal damage in glaucoma. Evidence supporting this hypothesis also arises from animal glaucoma models. Depositions of complement factor C3 and membrane attack complex (MAC) in the retina were found in a rat model of ocular hypertension (OHT) (Kuehn et al. 2006). MAC is able to build a pore in the membrane of the target cell, thereby causing osmotic stress and cell lysis (Bayly-Jones et al. 2017; DeLisi et al. 1980). In a rodent laser-photocoagulation induced OHT model, C3 split products and MAC levels were elevated. Complement depletion with cobra venom factor reduced retinal ganglion cell (RGC) loss (Jha et al. 2011). An activation of the complement cascade in early stages of the disease was also detected in the experimental autoimmune glaucoma model (Reinehr et al. 2016). Here, rats immunized with a bovine optic nerve antigen homogenate (ONA) developed a glaucomatous damage of the retina and the optic nerve without IOP elevation (Joachim et al. 2013; Laspas et al. 2011; Noristani et al. 2016). C3 and MAC depositions were detected in retinae and optic nerves of these animals, even before RGC death and axon demyelination. Besides, an increased number of microglial cells were found in these tissues in this model (Casola et al. 2016; Noristani et al. 2016). An activation of microglial cells before RGC death was also observed in a laser-induced rat OHT model (Ebneter et al. 2010). Furthermore, activated microglia occur in the optic nerve and retina in early stages of the disease in an inherited mouse model of glaucoma (Bosco et al. 2011), thus suggesting a potential role of microglia in glaucoma progression. Recently, the successful inhibition of the complement activation in the retinae via intravitreal administration of a C5 antibody in the autoimmune glaucoma model was reported (Reinehr et al. 2019a). The treatment resulted in a reduction of retinal MAC depositions, accompanied by a protection of RGCs and partial maintenance of the retinal function in electroretinography.

By now, the effects of a complement inhibition on the optic nerve in glaucoma have not been investigated. Therefore, this study aims to assess whether the intravitreal therapy with an antibody against complement factor C5 can reduce glaucomatous damage of the optic nerve in the autoimmune glaucoma animal model.

## Material and methods

### Animals

All experiments involving animals adhered to the ARVO statement for the use of animals in ophthalmic and vision research. All experiments were approved by the animal care committee of North Rhine-Westphalia, Germany.

Male Lewis rats (Charles River, Sulzfeld, Germany), 6 weeks of age, were used for the experiments and kept under environmentally controlled conditions with free access to chow and water. Detailed observations and health checks, including eye exams, were performed regularly.

### Immunizations with optic nerve homogenate

**Fig. 1:**
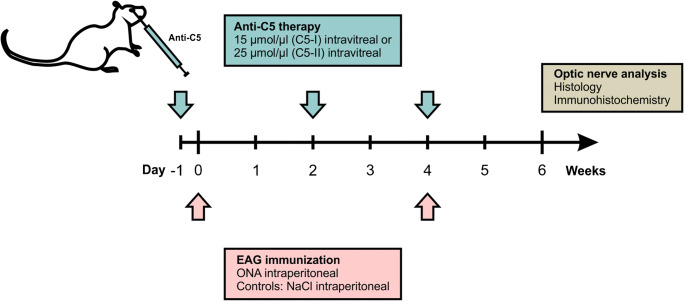
Study design. Animals were immunized with ONA at day zero. Immunization was boosted after 4 weeks. The intravitreal injections of the C5 antibody were performed 1 day before the first immunization (−1) and then repeated every 2 weeks (2 and 4 weeks). After 6 weeks, optic nerves were explanted, embedded, cut into longitudinal sections, and histologically examined

The rats were immunized with bovine optic nerve homogenate (ONA) as previously described (Laspas et al. 2011). The animals received intraperitoneal injections of 8 mg/ml ONA, mixed with 500 μl of incomplete Freund’s adjuvant plus 3 μg pertussis toxin (both Sigma-Aldrich, St. Louis, MO, USA) at day zero and with half this dose after 4 weeks (Fig. 1). The control group was treated with intraperitoneal injections of 0.9% sodium chloride in Freund’s adjuvant and pertussis toxin at the same points in time. To obtain the optic nerves, animals were sacrificed after 6 weeks by carbon dioxide inhalation.

### Intravitreal injections of C5 antibody

A monoclonal antibody against the complement factor C5 (BB5.1; Hycult Biotech, Uden, Netherlands) was injected intravitreally into one eye of some ONA immunized rats, as previously described (Reinehr et al. 2019a). For this, either a lower (15 μmol, *n* = 6; ONA + C5-I) or a higher (25 μmol, *n* = 6; ONA + C5-II) concentration of the antibody was applied. The control group was not treated intravitreally (*n* = 6). The anti-C5 therapy was first administered 1 day before the first immunization with ONA and was repeated every 2 weeks (Fig. 1).

For the injections, rats were anesthetized with a mixture of ketamine and xylazine (100/4 mg/kg). Mydriasis was induced by tropicamide 5% eye drops, and the ocular surface was locally anesthetized with oxybuprocaine-containing eye drops (Conjuncain, Bausch+Lomb, Rochester, NY, USA). The injection of either 5 or 8 μl BB5.1 was administered into the right eye of the rat under a stereomicroscope (Zeiss, Jena, Germany) using a 32 Gauge Hamilton cannula (Hamilton Company, Reno, NV, USA). An antibiotic eye ointment (Floxal, Bausch+Lomb) was applied onto the treated eye. The untreated left eyes of the animals constituted the ONA group (*n* = 6).

### Tissue preparation for histology and immunohistochemistry

The optic nerves were fixed in 4% paraformaldehyde for 2 h and dehydrated in a 30% sucrose solution. The tissues were then embedded in a cryostat matrix (Tissue Tek, Thermo Fisher, Waltham, CA, USA). Longitudinal sections of the optic nerves with a thickness of 4 μm were cut using a cryostat (Thermo Fisher) and were then mounted onto Superfrost microscope slides (Thermo Fisher).

### Optic nerve histology

#### Luxol fast blue staining

Longitudinal sections of the optic nerves (*n* = 6/group) were stained with luxol fast blue (LFB; RAL Diagnostics, Martillac Cedex, France) using a standard protocol to assess the axonal degeneration and myelin sheaths. Briefly, tissue sections were deparaffined with xylol and ethanol, dyed with warm LFB solution, and differentiated with lithium carbonate and ethanol before cover glasses were applied with Eukitt (O-Kindler GmbH, Freiburg, Germany). Microscopic photos were taken with the Axio Imager M1 (Carl Zeiss Microscopy, Oberkochen, Germany). The images were masked via AntRenamer software and then cut into equal excerpts of 800 × 600 pixels using Corel Paint Shop Pro X8 software (V18, Corel Corporation, Ottawa, Canada). A scoring system was used to determine the extent of demyelination in these (Renner et al. 2017). The images were scored from 0 (intact, combed structure of nerve fibers) to 2 (intense demyelination, wide holes) in 0.5 steps according to the degree of demyelination.

#### Hematoxylin and eosin staining

For the analysis of cellular infiltration of the optic nerve tissue, longitudinal sections were stained with hematoxylin and eosin (H&E; both Merck, Darmstadt, Germany; *n* = 6/group). The stained tissues were dehydrated in ethanol and then treated with xylene before cover glasses were applied onto microscope slides with Eukitt mounting medium. Three pictures of each optic nerve section were taken with a microscope (Axio Imager M1). The images were masked via AntRenamer software and then cut into equal excerpts of 800 × 600 pixels using Corel Paint Shop Pro X8 software. The arrangement of cell nuclei was evaluated, and the images of the H&E stained optic nerves were scored from 0 (no infiltrating inflammatory cells, bead-like arrangement of cell nuclei) to 4 (massive cellular infiltration) (Grotegut et al. 2019).

### Immunohistochemistry

For the assessment of complement components (C3 and MAC), (activated) microglia (Iba1 and ED1), macroglia (glial fibrillary acidic protein (GFAP)), and neurofilament (SMI-32), immunohistochemical stainings of the optic nerve sections were conducted (*n* = 5–6/group) (Reinehr et al. 2016). Six longitudinal optic nerve sections per animal were stained. In brief, the tissue sections were blocked in a solution of goat and/or donkey serum and 0.1 or 0.2% Triton-X in PBS for 1 h. The primary antibodies (Table 1) were added and incubated at room temperature overnight. The next day, the incubation with corresponding secondary antibodies was performed for 1 h (Table 1). Cell nuclei were stained with 4′,6 diamidino-2-phenylindole (DAPI; Serva Electrophoresis, Heidelberg, Germany). In order to obtain negative controls, the same staining protocols were carried out without primary antibodies.

**Table 1:** Primary and secondary antibodies applied for immunohistochemistry of optic nerve tissue

Primary antibodies			Secondary antibodies		
Antibody	Company	Dilution	Antibody	Company	Dilution
C3	Cedarlane	1:500	Goat anti-rabbit IgG Cy 3	Linaris	1:500
C5b-9 (MAC)	Biozol	1:100	Donkey anti-mouse Dy Light 488	Dianova	1:500
ED1	Millipore	1:250	Donkey anti-mouse Alexa Fluor 488	Dianova	1:500
GFAP	Millipore	1:500	Donkey anti-chicken Cy 3	Millipore	1:500
Iba1	Wako	1:500	Goat anti-rabbit IgG Cy3	Linaris	1:500
SMI-32	Biolegend	1:6000	Goat anti-mouse Alexa Flour 488	Invitrogen	1:400

### Histological examinations

The stained optic nerve sections were photographed with a fluorescence microscope (Axio Imager M1, Zeiss, Oberkochen, Germany) at a × 400 magnification. Six longitudinal sections of each optic nerve were stained, and three photos of each optic nerve section were taken (proximal, central, distal). The images were masked and then cut into equal excerpts of 800 × 600 pixels using Corel Paint Shop Pro X8 software. The cell counts as well as the measurement of the area and the intensity were performed in the myelinated part of the optic nerves.

C3^+^, MAC^+^, Iba1^+^, and ED1^+^ cells were counted with ImageJ software. In order to identify activated microglia, ED1^+^ signals were only counted when colocalized with Iba1 (Gramlich et al. 2011; Hendrickx et al. 2017; Ito et al. 1998).

The analysis of the area and the intensity of the macroglial marker GFAP was performed using an ImageJ macro (Reinehr et al. 2018). Therefore, the images were transformed into greyscale and background labeling was subtracted (rolling ball radius: 50 pixels). For each picture, appropriate lower and upper thresholds were determined. The average of the lower threshold values and the highest upper threshold were applied for the automated analysis (lower threshold: 9.2; upper threshold: 73). Via a macro, the percentage of GFAP^+^ area as well as the intensity per image were measured. For the group comparison, mean values of each optic nerve were calculated and transferred to Statistica (V13, DELL, Tulsa, OK, USA).

For the neurofilament assessment, the photos of the anti-SMI-32-stained optic nerve sections were classified by means of an established scoring system (Reinehr et al. 2018). The images were categorized from 0 (intact optic nerve structure, no retraction bulbs) to 2 (loss of structural integrity, many retraction bulbs) in steps of 0.5.

## Statistical analysis

Statistical analyses were performed using Statistica software. The groups were compared to each other by one-way ANOVA, followed by Tukey HSD post-hoc test. Data are presented as mean ± standard error (SEM). *p* values below 0.05 were considered statistically significant: * *p* < 0.05, ** *p* < 0.01, *** *p* < 0.001.

## Results

### Successful complement inhibition

**Fig. 2:**
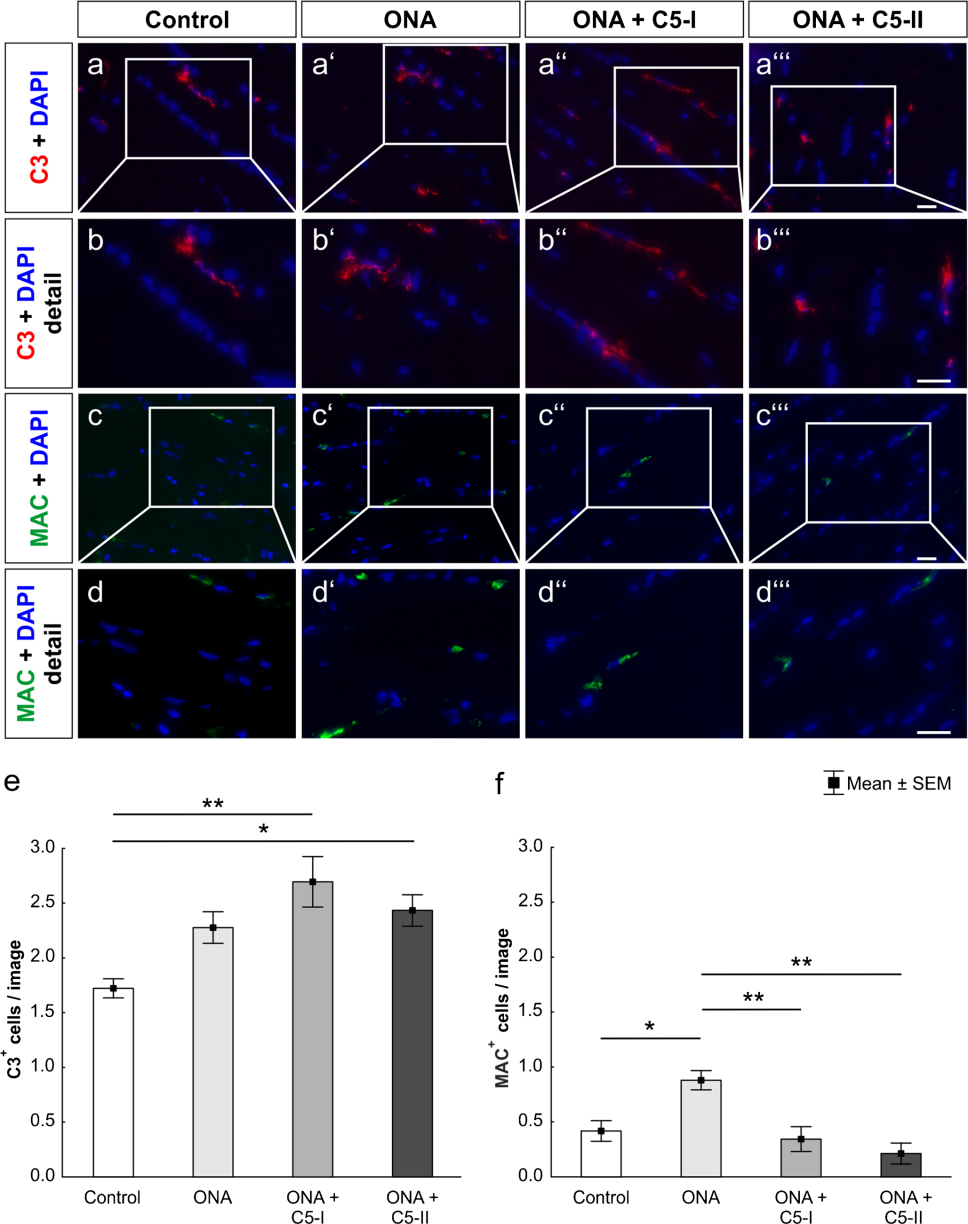
Successful complement inhibition. (a–a‴) Optic nerve sections were stained for complement factor C3 (red) DAPI (blue) was used to visualize cell nuclei. (b–b‴) Detailed pictures of C3 labeled optic nerves. (c–c‴) An antibody against the membrane attack complex (MAC, green) was used to label optic nerves, while DAPI (blue) counterstained cell nuclei. (d–d‴) Detailed images of optic nerves stained with MAC. (e) The amount of C3^+^ cells was significantly higher in the ONA + C5-I group (*p* < 0.01) and in the ONA + C5-II group (*p* < 0.05) compared to controls. (f) MAC^+^ cells were more frequent in the ONA group compared to controls (*p* < 0.05) and both treatment groups (both *p* < 0.01). Values are mean ± SEM. Scale bars: 20 μm

Regarding the ONA group, the amount of C3^+^ cells was not significantly different from controls (*p* = 0.10; Fig. 2 (a–a‴), (b–b‴), and (e); Table 2). In both intravitreally treated groups, significantly more C3^+^ cells were found compared to controls (ONA + C5-I: *p* = 0.002; ONA + C5-II: *p* = 0.04).

**Table 2:** Immunohistochemical and histological analyses of longitudinal optic nerve sections. Values are mean ± SEM. *p* values are indicated in relation to controls or ONA group. Significant *p* values are marked in italics

	C3^+^ cells/image	MAC^+^ cells/image	LFB score	SMI-32 score	H&E score	Iba1^+^ cells/image	ED1^+^ and Iba1^+^ cells/image	GFAP^+^ area (%)/image	GFAP intensity/image
Control	1.7 ± 0.1	0.4 ± 0.1	0.5 ± 0.04	0.2 ± 0.1	1.3 ± 0.1	0.7 ± 0.1	0.3 ± 0.1	1.3 ± 0.6	0.012 ± 0.0
ONA	2.3 ± 0.1	0.9 ± 0.1	0.9 ± 0.06	1.0 ± 0.1	2.4 ± 0.1	2.1 ± 0.2	1.2 ± 0.2	1.7 ± 0.5	0.015 ± 0.0
*p* value (vs control)	0.10	*0.01*	*0.01*	*< 0.001*	*0.001*	*< 0.001*	*0.01*	0.90	0.84
ONA + C5-I	2.7 ± 0.2	0.3 ± 0.1	0.6 ± 0.1	0.4 ± 0.1	1.4 ± 0.2	1.3 ± 0.2	0.7 ± 0.2	1.8 ± 0.2	0.012 ± 0.0
*p* value (vs control)	*0.002*	0.95	0.81	0.18	0.98	0.18	0.32	0.87	0.99
*p* value (vs ONA)	0.28	*0.005*	0.07	*< 0.001*	*0.003*	*0.03*	0.29	0.99	0.82
ONA + C5-II	2.4 ± 0.1	0.2 ± 0.1	0.7 ± 0.1	0.6 ± 0.02	2.0 ± 0.3	1.1 ± 0.2	0.6 ± 0.3	2.5 ± 0.6	0.017 ± 0.0
*p* value (vs control)	*0.04*	0.52	0.31	*0.001*	0.06	0.59	0.68	0.36	0.65
*p* value (vs ONA)	0.92	*0.002*	0.48	*0.002*	0.41	*0.009*	0.13	0.72	0.97

A significantly higher number of MAC^+^ cells was observed in ONA optic nerves compared to controls (*p* = 0.01) and both treatment groups (ONA + C5-I: *p* = 0.005; ONA + C5-II: *p* = 0.002; Fig. 2 (c–c‴), (d–d‴), and (f); Table 2). Between both therapy groups and controls no significant differences in MAC^+^ cells were detected (ONA + C5-I: *p* = 0.95; ONA + C5-II: *p* = 0.53). These results suggest a successful inhibition of the terminal complement pathway in the optic nerve via intravitreal C5 therapy, similar to recent findings in the retina (Reinehr et al. 2019a).

### Anti-C5 therapy reduces demyelination and preserves optic nerve structure

**Fig. 3:**
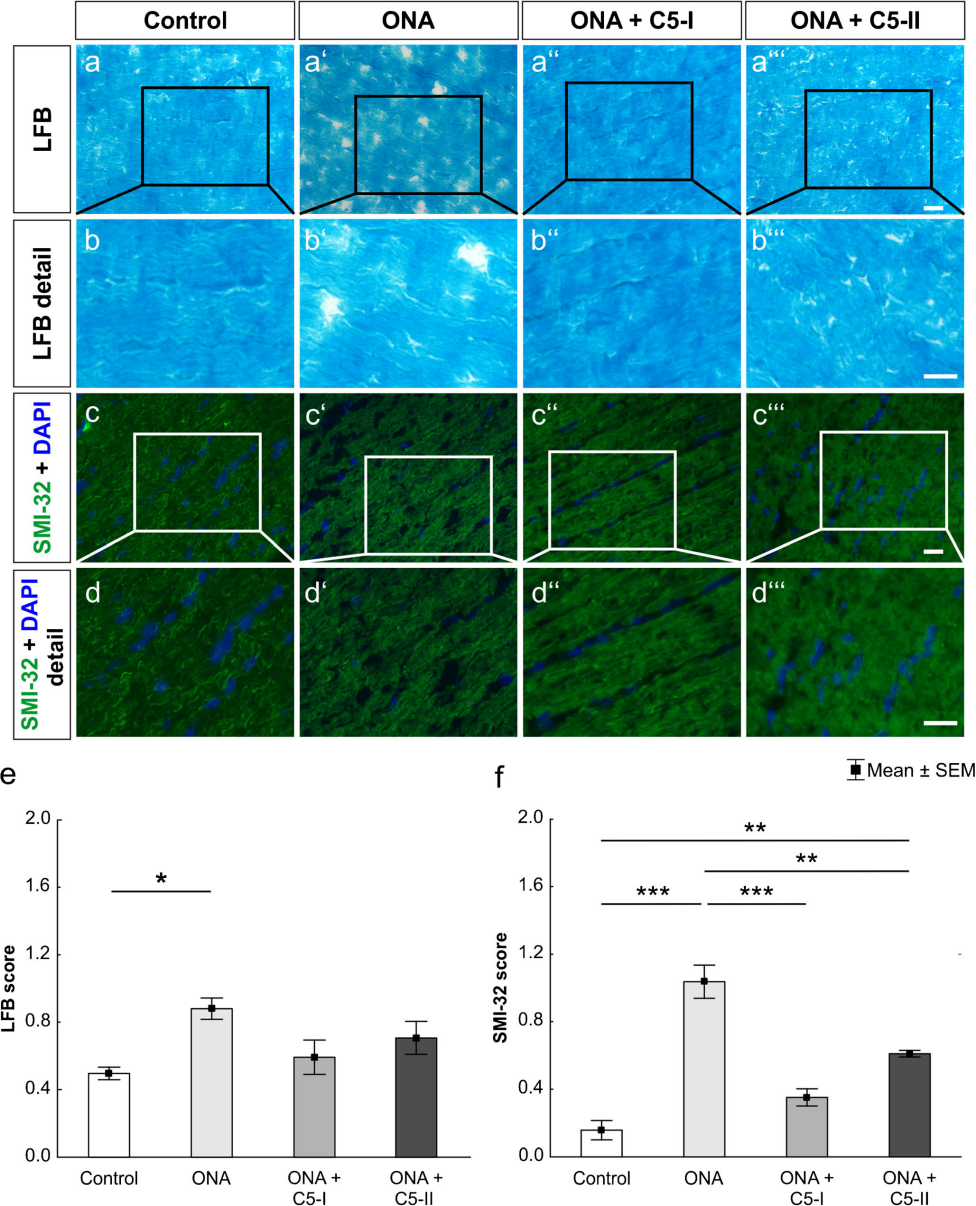
Preserved optic nerve structure and reduced demyelination. (a–a‴) Sections of the optic nerve were stained with Luxol Fast Blue (LFB). (b–b‴) Detailed pictures of LFB stained optic nerves. (c–c‴) An anti-SMI-32 antibody was used to label neurofilaments (green). Cell nuclei were visualized with DAPI (blue). (d–d‴) SMI-32 stained optic nerves shown in detail. (e) The highest LFB score values were reached in the ONA optic nerve compared to controls (*p* < 0.05). Scores in the treatment groups were not significantly increased. (f) Also, the highest SMI-32 score values were detected in the ONA group compared to controls (*p* < 0.001). Also, ONA + C5-I (*p* < 0.001) and ONA + C5-II optic nerves (*p* < 0.01) had higher scores. However, the ONA + C5-II group showed significantly higher score ratings than controls (*p* < 0.01). Values are mean ± SEM. Scale bars: 20 μm

Scoring of LFB-stained optic nerve sections revealed significantly higher score values in the ONA group in comparison to controls (*p* = 0.01; Fig. 3 (a–a‴), (b–b‴), and (e); Table 2). There were no significant differences between ONA + C5-I animals and controls (*p* = 0.81) or ONA + C5-II animals and controls (*p* = 0.31). Differences between the treated groups and ONA group were not statistically significant (ONA + C5-I: *p* = 0.07; ONA + C5-II: *p* = 0.48).

Concerning the SMI-32 score, the ONA group again showed the highest score values as well. The optic nerves of this group were scored significantly higher than controls (*p* < 0.001; Fig. 3 (c–c‴), (d–d‴), and (f); Table 2) and both ONA + C5-I (*p* < 0.001) and ONA + C5-II tissues (*p* = 0.002). The difference between the ONA + C5-I and the control group was not statistically significant (*p* = 0.18). In the ONA + C5-II optic nerves, a significantly higher SMI-32 score was detected compared to controls (*p* = 0.001). In summary, the optic nerves of the treated groups stayed at similar levels as the control groups, whereas the untreated ONA groups showed higher score values indicating greater structural damage.

### Diminished cellular infiltration

**Fig. 4:**
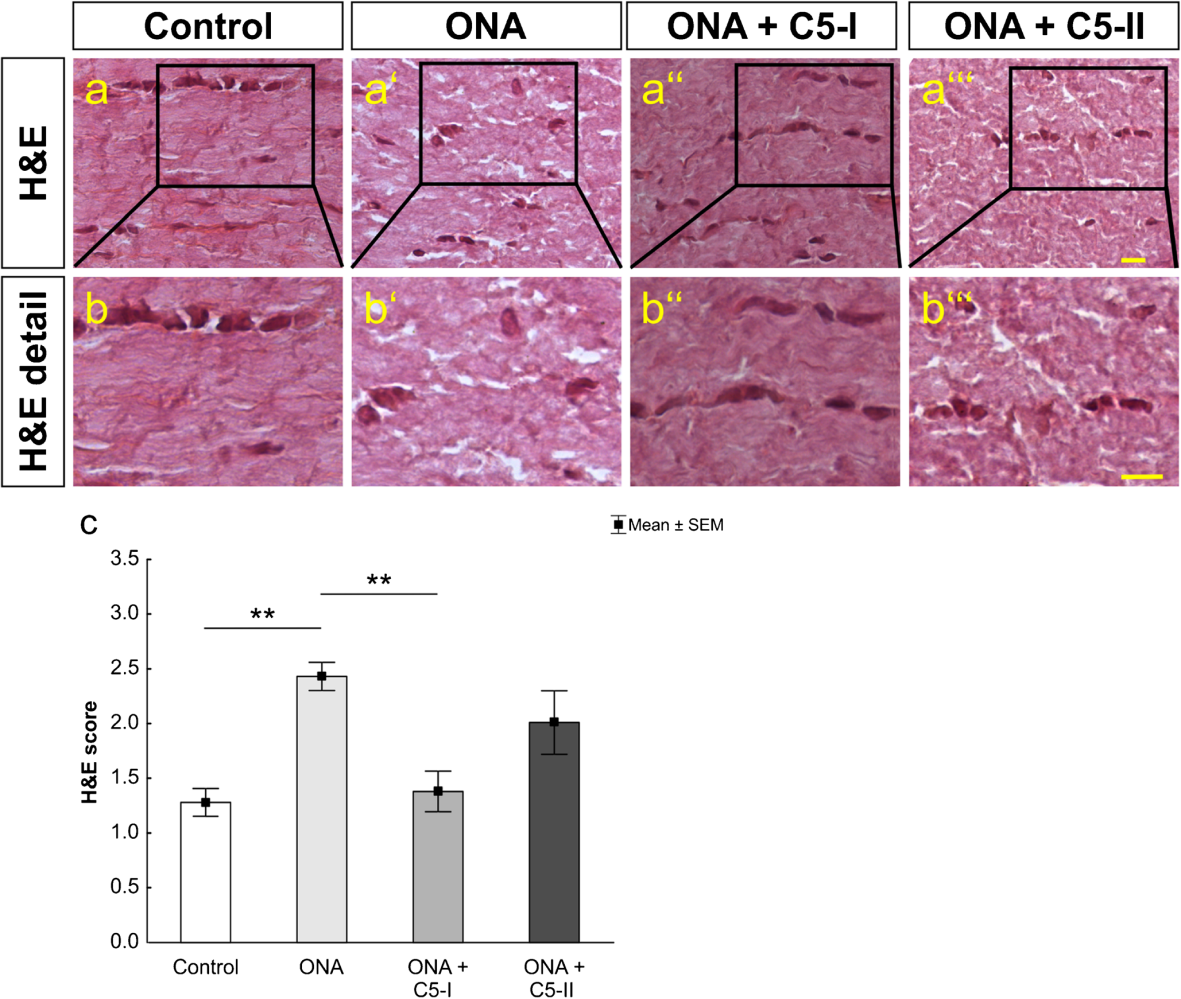
Less inflammatory cell invasion. (a–a‴) A histological staining of longitudinal optic nerve sections with H&E was performed. (b–b‴) Detailed pictures of H&E labeled optic nerves. (c) The ONA group displayed significantly higher H&E score values than controls (*p* < 0.01) and the ONA + C5-I group (*p* < 0.01). ONA + C5-II score values showed no significant differences to any other group. Values are mean ± SEM. Scale bars: 20 μm

The H&E stained optic nerves of the ONA group were scored significantly higher compared to the control (*p* = 0.001; Fig. 4 (a–a‴), (b–b‴), and (c); Table 2) and the ONA + C5-I group (*p* = 0.003). No significant differences were found between the treated groups and controls (ONA + C5-I: *p* = 0.98; ONA + C5-II: *p* = 0.06). Between ONA and ONA + C5-II, no significant difference was noted (*p* = 0.41). Thus, the intravitreal complement inhibition seems to prevent inflammatory cellular infiltration of the glaucomatous optic nerves.

### Decreased microglial activation

**Fig. 5:**
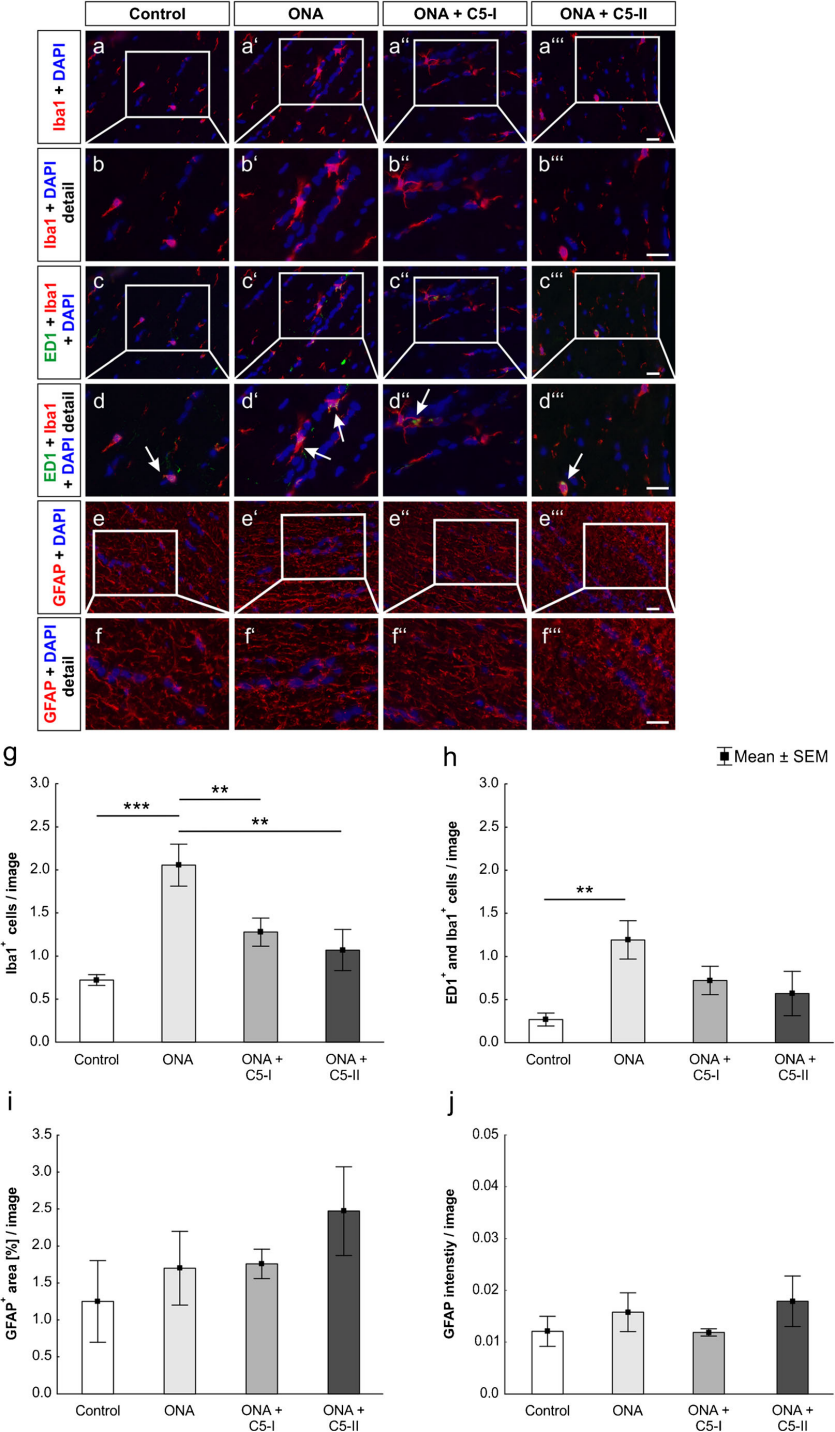
Decreased microglial activation. (a–a‴) Cells were labeled with anti-Iba1 for microglial cells (red) and DAPI (blue) for cell nuclei. (b–b‴) Detailed images of Iba1 stained optic nerves. (c–c‴) Iba1 (red) ín combination with the surface marker ED1 (green) identified activated microglia. Cell nuclei were stained with DAPI (blue). (d–d‴) In the detailed pictures, white arrows point to co-localizations of ED1 and Iba1. (e–e‴) Furthermore, anti-GFAP (red) was used to mark the cytoskeleton of macroglial cells. DAPI (blue) counterstained cell nuclei. (f–f‴) A detailed overview of GFAP labeled optic nerves. (g) The number of Iba1^+^ cells was significantly higher in the ONA group in comparison to the control (*p* < 0.001) and both therapy groups (both *p* < 0.01). (h) Activated microglia were more frequent in the ONA group compared to controls (*p* < 0.01). The differences between ONA + C5-I, ONA + C5-II, and Co were not significant. (i) Regarding the GFAP^+^ area, no significant differences could be detected between all groups. (j) Additionally, the intensity of GFAP was not altered within all groups. Values are mean ± SEM. Scale bars: 20 μm

The number of microglial (Iba1^+^) cells was significantly higher in the ONA group compared to controls (*p* < 0001; Fig. 5 (a–a‴), (b–b‴), and (g); Table 2) and both therapy groups (ONA + C5-I: *p* = 0.03; ONA + C5-II: *p* = 0.009). The number of microglial cells did not differ significantly between the control and therapy groups (ONA + C5-I: *p* = 0.18, ONA + C5-II: *p* = 0.59).

Regarding activated microglia, there were significantly more ED1^+^ and Iba1^+^ cells in the ONA group in comparison to controls (*p* = 0.01; Fig. 5 (c–c‴), (d–d‴), and (h); Table 2). The number of activated microglia remained unchanged in both therapeutic optic nerves compared to the control ones (ONA + C5-I: *p* = 0.32; ONA + C5-II: *p* = 0.68).

The analysis of the GFAP^+^ area did not demonstrate any significant alterations within all groups (*p* > 0.05; Fig. 5 (e–e‴), (f–f‴), and (i); Table 2). Furthermore, the intensity of GFAP was not altered between all groups (*p* > 0.05; Fig. 5 (e–e‴), (f–f‴), and (j); Table 2).

## Discussion

By now, glaucoma is one of the leading causes of blindness worldwide and occurs commonly in older people (EGS 2017, Weinreb et al. 2014). Considering the demographic change, the importance of efficient therapies will further increase (Weinreb et al. 2014). However, currently available treatments are insufficient since they focus only on IOP reduction and have serious side effects, thus leading to poor patient compliance (Gupta and Chen 2016). Further knowledge of the exact pathomechanisms of glaucoma is urgently needed to define novel targets for better therapeutic options for glaucoma patients, especially for those suffering from NTG.

### Contribution of the complement system in glaucoma

Besides of an elevated IOP, excitotoxicity (Dreyer et al. 1996; Lagreze et al. 1998; Vorwerk et al. 2000) and oxidative stress (Inman et al. 2013; Ko et al. 2005; Tezel et al. 2010; Yang et al. 2016) might matter for the development of glaucoma disease. Lately, the attention of glaucoma researchers shifted progressively to the participation of the immune system in the pathogenesis (Grus et al. 2008; Tezel and Wax 2004; Wax 2011; Yang et al. 2019). Both innate and adaptive immunity are implicated in this neurodegenerative disease (Wax and Tezel 2009). In particular, the complement system seems to contribute to disease onset and progression. It is part of the innate immunity and a key driver in inflammatory processes and immune defense against infections. Recently, it was shown that each retinal cell type itself expresses complement components (Pauly et al. 2019). However, a dysregulation of complement activation contributes to many autoimmune or degenerative and inflammatory diseases (Morgan and Harris 2015). In retinal protein samples of human donor eyes from glaucoma patients, an increased expression of complement factors was detectable via mass spectrometry and immunohistochemical analyses. Moreover, in in vitro experiments with rat retinal cell cultures, the complement factor H was downregulated (Tezel et al. 2010). The experimental inhibition of factor C1 prevented dendritic and synaptic atrophy of retinal ganglion cells (Williams et al. 2016). After IOP elevation, the activation of the complement system was noted besides RGC loss in a rat OHT model (Becker et al. 2015). Gene therapy with a C3-inhibitor resulted in neuroprotection of RGCs in DBA/2J mice (Bosco et al. 2018), while C5-deficient DBA/2J mice developed a less severe glaucomatous damage than their C5-sufficient counterparts (Howell et al. 2013). Former studies noted an activation of the complement system in the autoimmune glaucoma model (Reinehr et al. 2018; Reinehr et al. 2016). Furthermore, an improved survival of RGCs, inhibition of MAC formation, and ameliorated retinal function after intravitreal C5 antibody therapy have lately been reported in retinae after ONA immunization (Reinehr et al. 2019a). However, the possible effects of a complement inhibition on the glaucomatous optic nerve have not yet been investigated.

In this study, we analyzed the effects of this therapy on the optic nerve for the first time. Our results indicate that MAC formation was inhibited by the intravitreal treatment, whereas C3^+^ signals were increased in every ONA-immunized group and even significantly more frequent in the intravitreally treated ones. Since the cleavage of C3 is upstream of MAC formation in the cascade, it seems logical that only the pathways downstream of C5 are inhibited by the anti-C5 antibody. The complement system can be activated via three different pathways, the classical, the alternative, and the lectin pathway. All pathways lead to the cleavage of C3 into C3a and C3b as well as to the cleavage of C5 into C5a and C5b. C5b contributes to the formation of the membrane attack complex C5b9. This complex is able to build a pore in the membrane of target cells, thereby causing osmotic stress with increased intracellular calcium concentrations which induce apoptosis (Wang et al. 1999). Our results are similar to those of Copland et al. who showed that both systemic and local C5 inhibition with the monoclonal anti-C5 antibody BB5.1 reduced clinical manifestations of experimental autoimmune uveoretinitis (Copland et al. 2010).

### Protective effects on optic nerve structure

Glaucoma is characterized by an irreversible loss of optic nerve fibers, which results in visual field defects, culminating in blindness (Bowling 2016). In our study, LFB and SMI-32 staining revealed the most severe structural damage in the optic nerves of the ONA group, whereas the scores of the treated groups stayed within the level of controls. These results suggest a protection of neurofilament and myelin sheaths by intravitreal C5 inhibition. In addition, the H&E stained optic nerves of the ONA group displayed the strongest inflammatory cellular infiltration, while there were no significant differences between treated groups and controls. Hence, the treatment seems to prevent cellular infiltration. In a former study using the autoimmune glaucoma model, immunization with the glial protein S100B led to a significant damage of neurofilaments as observed in SMI-32-stained optic nerves. Furthermore, a tendency towards a progressive degeneration of LFB-stained optic nerve myelin sheaths was observed (Kuehn et al. 2018). Similarly, in another study, a time-dependent structural degeneration of myelin and neurofilament was found after immunization with ONA (Noristani et al. 2016). These findings are in accordance with our current results in the ONA group. The structural improvement we observed in the anti-C5 treated groups indicates potential therapeutic benefits of this treatment for glaucoma.

### Microglial response to intravitreal C5 inhibition

Regarding microglia, we detected a significant increase in cell numbers in the ONA group, but not in the C5 therapy optic nerves. Similar results were observed for the activated microglia. ED1^+^ and Iba1^+^ cells occurred significantly more frequently in optic nerves of ONA animals, but there were no significant alterations between the anti-C5 treated groups and controls. These findings suggest that the intravitreal therapy was able to diminish microglial response and activation in the optic nerve.

Consistent with our data, increased numbers of microglial cells were observed in mice 6 weeks after immunization with ONA (Reinehr et al. 2019b). An intravitreal injection of S100B, as part of ONA, led to significantly more activated microglia in optic nerves of rats already after 14 days (Grotegut et al. 2019). Also, in several OHT glaucoma animal studies, microglia were investigated. For example, Son et al. noted activated microglia in DBA2/J optic nerves (Son et al. 2010). Furthermore, activated microglia concentrated in the optic discs of DBA2/J mice in early stages of the disease (Bosco et al. 2011). After translimbal laser photocoagulation in rats, microglia hypertrophy in addition to retraction of microglia processes was observed after 3 days (Ebneter et al. 2010). All these studies indicate a contribution of microglia activation in optic nerve damage. Inhibition of microglia through minocycline or irradiation reduced axon degeneration in DBA2/J mice without IOP lowering (Bosco et al. 2012, 2008). Interestingly, after optic nerve crush injury in mice, the depletion of microglia via the colony stimulating factor 1 receptor inhibitor could not prevent optic nerve and retinal neurodegeneration (Hilla et al. 2017). In our study, not microglia but the complement system was inhibited. This raises the question how this inhibition could affect the number of microglia. In animal models of Alzheimer’s disease it could be shown that an administration of a C5a antagonist could attenuate activated microglia (Fonseca et al. 2009). Furthermore, the gene profile of microglia showed less inflammation and a higher induction of clearance pathways after the genetic ablation of C5aR1 in an Alzheimer’s disease mouse model (Hernandez et al. 2017). These data imply that the intravitreal complement inhibition could possibly decrease microglial response, thereby helping to prevent optic nerve degeneration in experimental glaucoma.

### Macroglial cells barely affected

Astrocytes are the most abundant cell type in the optic nerve head. Naturally, they support the axons, but after an injury or a disease, they become reactive (Sofroniew and Vinters 2010) and express more GFAP (Ridet et al. 1997). In glaucoma, astrocyte alterations probably have both beneficial and destructive effects on RGC survival (Johnson and Morrison 2009). Our experiments did not reveal any significant differences regarding the GFAP^+^ area between the groups. In the retinae of the autoimmune glaucoma model similar results were observed, since immunohistochemical analyses did not show any alterations in GFAP^+^ area. Nonetheless, RT-qPCR demonstrated an upregulation of *Gfap* mRNA levels in ONA as well as in treated retinae (Reinehr et al. 2019a). In former studies on the autoimmune glaucoma model in rats, only a slight macroglial response was observed after 4 weeks (Noristani et al. 2016; Reinehr et al. 2016). It is possible that in rats a gliosis occurs at later points in time due to different concentrations of ONA in rats and mice. An increase of GFAP could namely be detected in the optic nerves of mice 6 weeks after ONA immunization (Reinehr et al. 2019b). Independent from an elevated IOP, the intravitreal injection of S100B provoked an astrocyte response in the optic nerves of rats (Grotegut et al. 2019).

In an OHT model, a strong increase in astrocyte density of the optic nerve was found after laser-induced IOP elevation, correlating with the loss of axons (Mabuchi et al. 2003). An early increase of astrocyte reactivity was detected both in a rat OHT model and DBA/2 J mice (Son et al. 2010). In the *Meg2* HET model, mice develop IOP elevation as well as glaucomatous damage in retina and optic nerve. Here, a significant increase of GFAP^+^ staining area could be revealed in the optic nerves of the transgenic animals (Reinhard et al. 2019).

All these results indicate that an astrogliosis is more prominent after IOP elevation. Nonetheless, in the EAG model a reaction of astrocytes in the optic nerve could be detected at subsequent points in time.

## Conclusion

Our results point to the potentially beneficial effects of an intravitreal inhibition of the complement pathway on optic nerves in the autoimmune glaucoma model for the first time. Anti-C5 therapy reduced MAC formation, preserved optic nerve structure, and diminished inflammatory as well as microglia cells. Consequently, intravitreal C5 inhibition might be a future therapeutic approach for glaucoma.
